# Trajectories of Opioid Use Following First Opioid Prescription in Opioid-Naive Youths and Young Adults

**DOI:** 10.1001/jamanetworkopen.2021.4552

**Published:** 2021-04-22

**Authors:** J. Deanna Wilson, Kaleab Z. Abebe, Kevin Kraemer, Jane Liebschutz, Jessica Merlin, Elizabeth Miller, David Kelley, Julie Donohue

**Affiliations:** 1 Division of General Internal Medicine, Department of Medicine, University of Pittsburgh, Pittsburgh, Pennsylvania; 2 Division of Adolescent and Young Adult Medicine, Department of Pediatrics, University of Pittsburgh, Pittsburgh, Pennsylvania; 3 Pennsylvania Office of Medical Assistance Programs, Harrisburg; 4 Department of Health Policy and Management, University of Pittsburgh Graduate School of Public Health, Pittsburgh, Pennsylvania

## Abstract

**Importance:**

Although prescription opioids are the most common way adolescents and young adults initiate opioid use, many studies examine population-level risks following the first opioid prescription. There is currently a lack of understanding regarding how patterns of opioid prescribing following the first opioid exposure may be associated with long-term risks.

**Objective:**

To identify distinct patterns of opioid prescribing following the first prescription using group-based trajectory modeling and examine the patient-, clinician-, and prescription-level factors that may be associated with trajectory membership during the first year.

**Design, Setting, and Participants:**

This cohort study examined Pennsylvania Medicaid enrollees’ claims data from 2010 through 2016. Participants were aged 10 to 21 years at time of first opioid prescription. Data analysis was performed in March 2020.

**Main Outcomes and Measures:**

This study used group-based trajectory modeling and defined trajectory status by opioid fill.

**Results:**

Among the 189 477 youths who received an initial opioid prescription, 107 562 were female (56.8%), 81 915 were non-Latinx White (59.6%), and the median age was 16.9 (interquartile range [IQR], 14.6-18.8) years. During the subsequent year, 47 477 (25.1%) received at least one additional prescription. Among the models considered, the 2-group trajectory model had the best fit. Of those in the high-risk trajectory, 65.3% (n = 901) filled opioid prescriptions at month 12, in contrast to 13.1% (n = 6031) in the low-risk trajectory. Median age among the high-risk trajectory was 19.0 years (IQR, 17.1-20.0 years) compared with the low-risk trajectory (17.8 years [IQR, 15.8-19.4 years]). The high-risk trajectory received more potent prescriptions compared with the low-risk trajectory (median dosage of the index month for high-risk trajectory group: 10.0 MME/d [IQR, 5.0-21.2 MME/d] vs the low-risk trajectory group: 4.7 MME/d [IQR, 2.5-7.8 MME/d]; *P* < .001). The trajectories showed persistent differences with more youths in the high-risk trajectory going on to receive a diagnosis of opioid use disorder (30.0%; n = 412) compared with the low-risk group (10.1%; n = 4638) (*P* < .001).

**Conclusions and Relevance:**

This study’s results identified 2 trajectories associated with elevated risk for persistent opioid receipt within 12 months following first opioid prescription. The high-risk trajectory was characterized by older age at time of first prescription, and longer and more potent first prescriptions. These findings suggest even short and low-dose opioid prescriptions can be associated with risks of persistent use for youths.

## Key Points

**Question:**

What patterns of opioid prescribing exist following the first opioid prescription in a cohort of opioid-naive youths (aged 10-21 years)?

**Findings:**

In this cohort study including 189 477 youths, there were 2 distinct trajectories; and 65.3% of patients in the high-risk trajectory group filled opioids at 12 months compared with 13.1% in the low-risk trajectory. Differences between the 2 trajectories persisted beyond 12 months, with a greater proportion of both opioid fills and opioid use disorder diagnoses in the high-risk group.

**Meaning:**

Among the highest-risk trajectory, even short and low-dose opioid prescriptions were associated with increased risk of persistent opioid use.

## Teaser

This cohort study examines trajectories of opioid use among youths aged 10 to 21 years who had been prescribed opioids for the first time.

## Introduction

As 1 out of every 10 deaths among adolescents and young adults in the United States is a result of opioids,^1^ it is critical to examine the impact of prescription opioids as they remain the first exposure to opioids for many youths.^2^ Opioid prescribing remains high among pediatric patients,^3^ particularly in acute care settings, with nearly 14.9% of youths receiving opioids during emergency department visits.^4^

Youths may have higher risk from early opioid exposure as the developmentally immature brain places greater salience on substance use–related rewards and has decreased ability to moderate substance use or anticipate substance use–related consequences.^5,6^ Evidence suggests that the earlier the age of first opioid prescription, the greater the risk for developing opioid misuse or illicit opioid use.^7^ Long-term opioid therapy (receiving additional opioid prescriptions following the first index opioid prescription) has limited evidence to support such use for managing pain in adults,^8^ and even less in youths.^9^ Long-term opioid therapy, defined as greater than 90 days of opioid use, confers additional risks for opioid misuse^10^ and an added risk of developing opioid use disorder (OUD), a clinical diagnosis defined by the *Diagnostic and Statistical Manual of Mental Disorders* (Fifth Edition) as a spectrum of problematic, compulsive use associated with increasingly negative consequences.^11^

More than half of adults with a substance use disorder, including OUD, report that symptoms started before the age of 18, and the majority of individuals (80%) reported onset of symptoms by the age of 24 years.^12^ Multiple studies support prescription opioid use as a key risk factor in development of opioid misuse or illicit opioid use among youths.^2,13,14,15^ Although the vast majority of youths prescribed opioids do not go on to report opioid misuse,^14^ most of those with a history of illicit heroin use or prescription opioid misuse report their initial opioid use was a prescription from a health care practitioner.^7,13,16^ Although we know opioid prescribing during adolescence is an important risk factor for future opioid use, we currently lack an understanding of (1) how patterns of opioid prescribing at and following the first opioid exposure relate to persistent opioid use; and (2) factors associated with persistent opioid use for the 12 months following the first opioid prescription.

This study used claims data to study a cohort of children, adolescents, and young adults (aged 10 to 21 years at the time of their first opioid prescription) for 12 months following the first opioid prescription. We aimed to identify distinct patterns of opioid fills following the initial prescription using group-based trajectory modeling. We also examined the patient-, clinician-, and prescription-level factors associated with trajectory membership during the first year.

## Methods

### Study Design

We conducted a cohort study using enrollment and claims data from 2007 to 2016 for Pennsylvania Medicaid enrollees. This study was deemed exempt by the University of Pittsburgh institutional review board and had a waiver of informed participant consent because it was not considered human participants research. We presented findings according to the Strengthening the Reporting of Observational Studies in Epidemiology (STROBE) reporting guideline for cohort studies.

### Participants and Eligibility Criteria

Eligibility criteria for the study cohort included any Pennsylvania Medicaid enrollees who received at least one opioid prescription between the ages of 10 to 21 years. Opioid prescriptions were identified using pharmacy claims and National Drug Codes for both short- and long-term opioids (excluding methadone, which is unlikely to be used for pain control among opioid-naive individuals, and buprenorphine, which is primarily used to treat opioid use disorder). We defined the date of the opioid prescription (or first prescription in enrollees with multiple prescriptions) as the index date. Individuals were excluded from the cohort if they were eligible for long-term care or dual-eligible for Medicare or had less than 6 months of continuous enrollment in Medicaid prior to the index prescription. For individuals with multiple periods of opioid use in the claims data, we included only the first period of opioid use. All enrollees who received the index opioid prescription and at least one other opioid prescription during the subsequent year were included in the trajectory models. We further restricted the trajectory analyses to those with 12 months of continuous enrollment following index prescription (eFigure 1 in the Supplement). Sample size for logistic regression included only enrollees with all available covariate and variable data.

### Study Variables

#### Participant Characteristics

We used enrollment data to record race and ethnicity, collapsed into 4 categories: non-Latinx White, non-Latinx Black or African American, Latinx, and other races. We categorized Medicaid enrollment in either fee-for-service or a managed care organization based on status for majority of days within a 12-month period. We used *International Classification of Diseases, Ninth Revision (ICD-9)* and *International Classification of Diseases, Tenth Revision (ICD-10)* codes to define depression, anxiety, attention-deficit/hyperactivity disorder (ADHD), and substance use disorders (eg, cannabis use disorder, alcohol use disorder) in the year following the initial prescription using inpatient, outpatient, and professional claims (eTable in the Supplement). We defined cancer as a dichotomous variable and included individuals having ever received a cancer diagnosis, excluding nonmelanoma skin cancers, prior to the index opioid prescription.

#### Index Opioid Prescription and Prescribing Clinician

The month of the first opioid prescription was identified as the index month. Pharmacy claims were used to calculate the morphine milligram equivalents (MME) of the index opioid prescription and total days’ supply. This was used to calculate proportion of days covered in the index month and mean daily MME during the index month to calculate mean daily exposure during the index month. For enrollees who received multiple opioid prescriptions at the time of the first opioid prescription, the MME were summed to represent a total index prescription dose and days’ supply. As some enrollees received more than one opioid prescription during the index month, we created a dichotomous variable (yes or no) to code for receiving multiple prescriptions during the index month. We used the specialty clinician information from pharmacy claims to link to the National Plan and Provider Enumeration System to identify specialty of clinicians. We grouped clinicians into 7 categories by specialty: general medicine (including family medicine, internal medicine, general practitioners), dentistry (dentists and oral surgeons), emergency medicine, obstetrics and gynecology, pediatrics, surgical care, and other (such as ophthalmology or podiatry).

#### Primary Outcome to Define the Trajectory Groups

The primary outcome used to define the trajectory groups was opioid fills during the first year of the index prescription. We defined this using pharmacy claims. Enrollees were classified with binary indicators (based on yes or no for opioid fills for a given month).

### Statistical Analyses

#### Defining Trajectories

We first used group-based trajectory modeling to identify patterns of opioid prescription fills in the first year following an opioid prescription. We included individuals who received 2 or more opioid prescriptions within the first year and classified them into unique longitudinal polynomial trajectories based on opioid prescription fills. Models were tested from 2-group to 6-group models and prescription fill status as a smooth function of time. We determined the number of groups and degree of polynomial in each of the trajectory groups using bayesian information criterion (BIC) to measure improvement in model fit. Higher group models defaulted to 4-groups with 0% membership status in the fifth or higher group. The degree of the fitted polynomial was determined by examining all possible permutations up to 4th order polynomials. The best model was chosen using comparisons of estimated and observed adherence trajectories as well as BIC and clinical relevance. We conducted a sensitivity analysis excluding individuals with cancer. As the study examines trajectories for all youths prescribed opioids regardless of associated diagnoses, we reported findings for the entire sample. There were minimal changes in trajectory membership (n=8) when we excluded individuals with cancer.

#### Describing Trajectories

We used general linear models to compare means across different trajectories examining patient factors, prescriber specialty type, and first prescription characteristics. We examined differences between trajectories that persisted beyond 12 months by comparing mean opioid fills, mean months of enrollment, and proportion of opioid use disorder diagnoses using all available Medicaid claims data for each enrollee (beyond the 12 months that defined the trajectory).

#### Estimating Trajectory Membership

We used multinomial logistic regression models to calculate adjusted odds for trajectory membership based on patient, clinician, and prescription characteristics. Models were adjusted for age at index prescription, race/ethnicity, gender, patient comorbidities over the first year (such as cancer status, depression, anxiety, ADHD, or substance use disorders), clinician specialty type, and days’ supply and MME per day during index month and year of index prescription.

We used general linear models to compare means across different trajectories (significance threshold set at *P* < .05) examining patient factors, prescriber specialty type, and first prescription characteristics. Data analyses were performed using SAS version 9.4 (SAS Institute) in March 2020. We used the PROC TRAJ plug-in for SAS to create the trajectories.

## Results

### Cohort

Among the 189 477 youths who received an initial opioid prescription, the median age at first prescription was 16.9 years (interquartile range [IQR], 14.6-18.8 years), 107 562 were female (56.8%), and 112 911 (59.6%) were non-Latinx White (59.6%). Enrollees had high rates of mental illness, with 24 637 receiving a diagnosis of depression (13%), 25 681 receiving a diagnosis of ADHD (14%), and 10 719 receiving a diagnosis of anxiety (6%). The median index prescription was short (4.0 days duration [IQR, 3.0-5.0 days]) and relatively low mean (SD) daily dosage (57.7 [31.59] MME). During the first year, 10 985 youths received a diagnosis of OUD.

### Model Selection

There were 47 477 youths (25.1%) who filled at least one additional opioid prescription in the year following the first opioid fill and were used to define trajectories. Model fit indicated by BIC improved with additional trajectory groups: 1-group model (BIC = −230826.3), 2-group model (BIC = −223156.3), 3-group model defaulted to the 2-group model with 0% membership in 3rd group (BIC = −223183.2), and 4-group model (BIC = −218750.2). We present the 2-group model based on BIC (as the more parsimonious model). We fit the final model to quadratics as it was associated with best fit (Figure). The 4-group model findings are in eFigure 2 in the Supplement.

**Figure.**
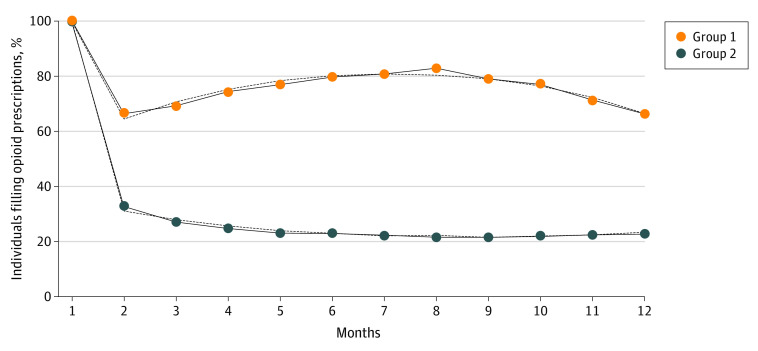
Trajectories of Opioid Fills During 12 Months Following First Opioid Prescription Dotted lines represent estimated trajectories and solid lines represent observed trajectories.

### Describing Trajectory Groups

Group 1 had 1380 enrollees (3.1% of the sample) and was defined as the high-risk trajectory based on 901 youths (65.3%) in this group filling opioid prescriptions at month 12. In contrast, group 2, which had 46 097 enrollees (96.9% of the sample), was defined as the low-risk trajectory, with 6031 youths (13.1%) still filling opioids at month 12 (Figure).

### Describing Trajectories Based on Characteristics

#### Patient Factors

There were age differences between the 2 trajectories: median age among the high-risk trajectory was 19.0 years (IQR, 17.1-20.0 years) compared with the low-risk trajectory (median age 17.8 years [IQR 15.8-19.4 years]) (difference = 0.93; 95% CI, 0.80-1.05; *P* < .001). The high-risk trajectory also had a greater proportion of non-Latinx White enrollees (1005 enrollees [72.8%]) compared with the low-risk group (29 474 enrollees [63.9%]) (difference = 0.09; 95% CI, 0.07-0.11; *P* < .001) and those who filled only the first index prescription (82 432 enrollees [58.1%]) (difference = 0.15; 95% CI, 0.13-0.17; *P* < .001). A greater proportion of enrollees in the high-risk trajectory had depression (369 enrollees [27.3%]) compared with the low-risk group (8074 enrollees [17.8%]) (difference in means = 0.09; 95% CI, 0.07-0.12; *P* < .001) or compared with those who filled only the index prescription (16 194 enrollees [11.8%]) (difference = 0.15; 95% CI, 0.13-0.18; *P* < .001). A greater proportion of those in the high-risk trajectory also had a diagnosis of cancer (103 enrollees [7.5%]) compared with the low-risk trajectory (1298 enrollees [2.8%]) (difference = 0.05; 95% CI, 0.03-0.06; *P* < .001) or compared with those who only filled one index opioid prescription (2392 enrollees [1.7%]) (difference = 0.06; 95% CI, 0.05-0.07; *P* < .001).

#### Prescription Factors

Although the index opioid prescription was short for all groups, the high-risk trajectory received longer prescriptions compared with the low-risk trajectory (median index opioid prescription length for high-risk trajectory group: 5.0 days [IQR, 3.0-12.0 days] vs low-risk trajectory group: 4.0 days [IQR, 3.0-5.0 days]; *P* < .001). The high-risk trajectory group also had more potent prescriptions (median dosage of the index month for high-risk trajectory group: 10.0 MME/d [IQR, 5.0-21.2] vs the low-risk trajectory group: 4.7 MME/d [IQR, 2.5-7.8]; *P* < .001) (Table 1).

**Table 1.** Descriptive Enrollee Characteristics by Trajectory Group for 2-Group Model

Measure	Enrollees, No. (%)				*P* value^a^	
	Overall	Received only index opioid prescription (single prescription)	Received ≥2 opioid prescriptions		Comparing 2-group model	Comparing 2 groups and single prescription
			Group 1 (high risk)	Group 2 (low risk)		
Enrollees	189 477 (100)	142 000 (74.9)	1380 (3.1)	46 097 (96.9)	NA	NA
Demographic factors						
Age at index prescription, median (IQR)	16.9 (14.6-18.8)	16.6 (14.2-18.5)	19.0 (17.1-20.0)	17.8 (15.8-19.4)	<.001	<.001
Race/ethnicity						<.001
Non-Latinx					<.001	<.001
White	112 911 (59.6)	82 432 (58.1)	1005 (72.8)	29 474 (63.9)		
Black	47 381 (25.0)	36 510 (25.7)	258 (18.7)	10 613 (23.0)		
Latinx	22 220 (11.7)	17 409 (12.3)	83 (6.0)	4728 (10.3)		
Other	6965 (3.7)	5649 (4.0)	34 (2.5)	1282 (2.8)		
Female	107 562 (56.8)	77 175 (54.3)	904 (65.5)	29 483 (64.0)	.24	<.001
Male	81 915 (43.2)	64 825 (45.7)	476 (34.5)	16 614 (36.0)		
MCO^b^	134 677 (71.1)	104 229 (73.4)	798 (57.8)	29 650 (64.3)	<.001	<.001
Patient comorbidities^c^						
Diagnosis within 12 mo						
Depression	24 637 (13.4)	16 194 (11.8)	369 (27.3)	8074 (17.8)	<.001	<.001
Anxiety	10 719 (5.8)	7306 (5.3)	140 (10.3)	3273 (7.2)	<.001	<.001
ADHD	25 861 (14.0)	20 090 (14.6)	141 (10.4)	5630 (12.4)	.03	<.001
Cancer	3793 (2.0)	2392 (1.7)	103 (7.5)	1298 (2.8)	<.001	<.001
Any diagnosis of opioid use disorder	10 985 (5.9)	5935 (4.3)	412 (30.0)	4638 (10.1)	<.001	<.001
Prescription characteristics						
Total supply for index prescription, median (IQR), d	4.0 (3.0-5.0)	4.0 (3.0-5.0)	5.0 (3.0-12.0)	4.0 (3.0-5.0)	<.001	<.001
Multiple opioid prescriptions during index month	1075 (0.6)	691 (0.5)	32 (2.3)	352 (0.8)	<.001	<.001
MME per day during index month, median (IQR)	4.5 (2.5-7.5)	4.2 (2.5-7.2)	10.0 (5.0-21.2)	4.7 (2.5-7.8)	<.001	<.001
Prescriber characteristics						
Prescriber type						
Dentistry	50 908 (37.7)	40 259 (39.9)	101 (10.0)	10 548 (31.8)	<.001	<.001
Emergency medicine	22 476 (16.6)	16 038 (15.9)	150 (14.9)	6288 (19.0)		
General medicine	29 170 (21.6)	20 303 (20.1)	478 (47.5)	8389 (25.3)		
Ob-gyn	7176 (5.3)	4951 (4.9)	69 (6.9)	2156 (6.5)		
Other	3696 (2.7)	2580 (2.6)	63 (6.3)	1053 (3.2)		
Pediatrics	4573 (3.4)	3333 (3.3)	80 (7.9)	1160 (3.5)		
Surgical care	17 087 (12.6)	13 474 (13.3)	66 (6.6)	3547 (10.7)		

Abbreviations: ADHD, attention-deficit/hyperactivity disorder; IQR, interquartile range; NA, not applicable; MCO, managed care organization; MME, milligram morphine equivalents; Ob-gyn, obstetrics and gynecology.

^a^ *P* values generated for each variable by separate, crude general linear models comparing means across different groups.

^b^ MCO or fee-for-service status coded based on status for majority of days within 12-month period.

^c^ Comorbidities defined as diagnosed within first 12 months of index prescription. Cancer defined by relevant *ICD-9* or *ICD-10* codes and could include historical or current cancer diagnosis.

### Persistent Differences Between Trajectories Beyond 1 Year

Examining trajectories beyond the first year showed no differences in total enrolled months between the groups, however, there were significant differences in the number of months with opioid fills (eg, the high-risk trajectory group with a mean [SD] of 26.5 [20.0] months compared with the low-risk trajectory group with 6.1 [7.5] months of opioid fills; difference in means = 20.3; 95% CI, 19.3, 21.4; *P* < .001). A greater proportion of youths in the high-risk trajectory received a diagnosis of opioid use disorder (30.0% [412 enrollees]) compared with the low-risk trajectory (10.1% [4638 enrollees]) (difference = 0.20; 95% CI, 0.17-0.22) or those who received only single index opioid enrollees (4.3% [5935 enrollees]) (difference = 0.26; 95% CI, 0.23-0.28; *P* < .001).

### Estimating Membership in a Given Trajectory

Compared with the low-risk trajectory, each additional year of age at time of first opioid prescription was associated with an adjusted 19% higher odds of being in the high-risk group (adjusted odds ratio [aOR], 1.19; 95% CI, 1.15-1.232). Black enrollees were associated with 38% lower odds of being in the high-risk group compared with non-Latinx White enrollees (aOR, 0.62; 95% CI, 0.52-0.75). Although female enrollees had lower odds of receiving only one prescription compared with male enrollees (aOR, 0.77; 95% CI, 0.75-0.79), they were equally likely to be in either trajectory (aOR, 0.91; 95% CI, 0.79-1.05) (Table 2).

**Table 2.** Final Adjusted Multinomial Logistic Regression Model Estimating Group Trajectory Membership for 2-Group Model^a^

Variable	Adjusted odds ratio (95% CI)		
	Single prescription	Group 1 (high risk)	Group 2 (low risk)
Age at time of index prescription	0.85 (0.85-0.86)	1.19 (1.15-1.23)	1 [Reference]
Race and ethnicity (non-Latinx, Black vs non-Latinx, white)	1.29 (1.25-1.33)	0.62 (0.52-0.75)	1 [Reference]
Gender (female vs male)	0.77 (0.75-0.79)	0.91 (0.79-1.05)	1 [Reference]
Cancer	0.63 (0.58-0.69)	2.07 (1.60-2.68)	1 [Reference]
Mean days’ supply for index prescription	0.97 (0.97-0.98)	1.09 (1.08-1.10)	1 [Reference]
Mean MME/d during index month	1.00 (0.999-1.00)	1.001 (1.001-1.002)	1 [Reference]
Depression	0.77 (0.74-0.80)	1.35 (1.16-1.59)	1 [Reference]
Anxiety	0.85 (0.80-0.89)	1.28 (1.02-1.60)	1 [Reference]
ADHD	0.97 (0.93-1.01)	0.81 (0.65-1.02)	1 [Reference]
Substance use disorder	0.75 (0.71-0.79)	1.09 (0.87-1.36)	1 [Reference]
Clinician type (dentistry vs general medicine)^b^	1.40 (1.35-1.45)	0.28 (0.22-0.35)	1 [Reference]
Year of index prescription (2015 vs 2007)^b^	2.17 (2.03-2.31)	0.62 (0.44-0.87)	1 [Reference]

Abbreviations: ADHD, attention-deficit/hyperactivity disorder; MME, milligram morphine equivalents.

^a^ Adjusted models included all variables within the table (including age at time of index prescription, race and ethnicity, gender, cancer, mean days’ supply, MME/d, depression, anxiety, ADHD, substance use disorder, clinician type, year of index prescription [as categorical variable]). Multinomial logistic regression models to calculate odds for trajectory membership based on patient, clinician, and prescription characteristics.

^b^ Additional group comparisons (eg, surgical care vs general medicine, 2013 vs 2008) available upon request. Model fit statistics included Akaike information criterion: 158144.74; Schwarz criterion: 158164.31; −2 log L: 158140.74); *R*^2^: 0.0738.

There were approximately twice the odds of being in the high-risk group if an enrollee currently or previously had cancer compared with the low-risk group (aOR, 2.07; 95% CI, 1.60-2.68). Depression and anxiety increased the risk for being in the high-risk group compared with the low-risk group (odds of being in the high-risk group if diagnosis of depression: aOR, 1.35; 95% CI, 1.16-1.59 or if diagnosis of anxiety: aOR, 1.28; 95% CI, 1.02-1.60).

If enrollees received a prescription from a dentist compared with a general medicine clinician, they had 72% lower odds of being in the high-risk trajectory (aOR, 0.28; 95% CI, 0.22-0.35). Each increase in length of index prescription by one day was associated with 9% increased odds of being in the high-risk trajectory (aOR, 1.09; 95% CI, 1.08-1.10) (Table 2).

## Discussion

This cohort study describes a large sample of young Medicaid enrollees, aged 10 to 21 years, following their first opioid prescription. In contrast with previous studies focusing on population-level risks following an initial opioid prescription,^14,15,17^ our study identified unique longitudinal trajectories of opioid fills during the year following the first opioid prescription. We identified a group of youths at persistent risk for filling opioid prescriptions 12 months following the first prescription. As prescription opioid use increases risk of opioid misuse^13,14,15,16^ and opioid misuse increases the risk for heroin initiation among adolescents,^18^ these findings have substantial public health implications. The persistently high rates of prescription opioid use identifies young people at elevated risk for future misuse, opioid-related morbidity and mortality, and development of OUD.^19,20,21^

The highest-risk trajectory, defined by risk of still filling opioid prescriptions at one year, was associated with a 3-fold higher proportion of youths diagnosed with OUD (or older definitions of opioid dependence or opioid abuse used in *Diagnostic and Statistical Manual of Mental Disorders* [Fourth Edition]) compared with those receiving only one opioid prescription. As OUD is frequently underdiagnosed by clinicians,^22^ this may be an underestimate of the true prevalence of OUD within this population. Clinicians may also be using a diagnosis of opioid dependence to identify patients with physiological dependence and not problematic use meeting current criteria for opioid use disorder. Clinicians seeing youths receiving persistent opioids (regardless of indication) should conduct a thorough assessment of potential mental health comorbidities, as well as screen for OUD. As young people have low rates of uptake of evidence-based medical therapies to treat OUD and gender and racial disparities exist in access to opioid use disorder treatment,^23^ routine screening for persistent opioid use during the first year following a prescription may improve early diagnosis and linkage to treatment.

Similar to previous research showing that depression is associated with an increased risk for persistent opioid use,^24^ we found a higher proportion of youth with depression in the highest-risk trajectory. Prolonged pain warranting opioids may increase the risk for developing depression, but the presence of depressive symptoms may also lead to impaired coping and thus unnecessary opioid use. Studies have found increased affective symptoms were associated with increased risk for opioid misuse^25^ and depressive symptoms were associated with increased odds for receiving an OUD diagnosis among those with prescription opioid misuse.^24^ Screening for depression or depressive symptoms at both time of first opioid prescribing and at subsequent visits may allow clinicians to recognize young people who may be at elevated risk for persistent opioid use or may identify those who developed depression as a result of pain.

There is less known about the impact of the first prescription on subsequent opioid use. Studies suggest substantial variation in index prescription duration and dosage,^26^ with longer prescriptions given more frequently to younger patients or to those seen in urgent care settings, but it is unclear how these factors may relate to subsequent misuse or persistent opioid prescribing.^27^ Our findings show longer prescriptions were associated with greater odds of receiving persistent opioid fills at 12 months than shorter, more potent prescriptions; however, the mean index prescription length even among the high-risk trajectory was still modest. In response to the opioid epidemic, some states have adopted prescribing regulations restricting days of supply for opioid prescriptions for minors to 7 days or less depending on the state,^28^ however, our findings would suggest even short, relatively low potency opioid prescriptions may not be without risk.

### Limitations

Our study has a number of limitations. Our sample includes data from Pennsylvania Medicaid and so represents a low-income population in a state hard hit by the opioid epidemic and may not be generalizable to other regions. Also, historical data may not reflect current prescribing patterns. As we only included those with continuous Medicaid enrollment for 6 months prior to and 12 months after the initial opioid prescription, we miss including those who disenrolled from Medicaid. Although we have identified prescriber specialty using the claims data, we are limited in the ability to link index prescriptions to specific diagnoses. Our sample also has a high level of missingness for specialty clinician, which may limit the generalizability to all clinicians. In addition, as we are limited to claims data, we are unable to identify those who actually consumed the opioids or those who paid for subsequent opioid prescriptions out-of-pocket or through other coverage, nor can we capture those who may not actually be opioid-naive as they received their first opioid exposure through alternative means (eg, self-pay or illicit means). The high rates of OUD diagnoses among those in the highest risk trajectory groups may reflect the presence of an OUD that was previously unrecognized (and led to the enrollee seeking the first opioid prescription in the claims data) as opposed to the development of a new disorder. ICD codes are a simplistic way of identifying diagnoses leading to both overdiagnosis and underdiagnosis. The small group size in the high-risk trajectory also may limit its generalizability to the sample as a whole. Regardless, membership within the highest-risk trajectories suggest a group requiring intensive screening and potential linkage to other treatment options.

## Conclusions

Using Medicaid claims data, we were able to identify distinct trajectories of opioid fills in the year following the first opioid prescription in a cohort of children, adolescents, and young adults. Although the highest-risk trajectory represents the smallest proportion of patients, it highlights a group with elevated risk of both persistent opioid use over the first year and also high rates of youths who go on to be diagnosed with OUD. Clinicians should screen for known comorbidities, such as depression, at the time of the first opioid diagnosis and throughout the first year among those with persistent opioid use. Clinicians should be thoughtful in minimizing opioid exposure and managing risk for those young people who appear to develop persistent opioid use early within the first year.

## Notes

Supplement. **eFigure 1.** Cohort Creation for Trajectory Models **eTable.** ICD 9 and 10 Codes Used to Define Patient Comorbidities **eFigure 2.** Four-Group Trajectory Model Showing Opioid Fills in 12 Months Following First Opioid Prescription

## References

[1] Gomes T, Tadrous M, Mamdani MM, Paterson JM, Juurlink DN. The burden of opioid-related mortality in the United States. JAMA Netw Open. 2018;1(2):e180217. doi:10.1001/jamanetworkopen.2018.0217 30646062 PMC6324425

[2] McCabe SE, West BT, Veliz P, McCabe VV, Stoddard SA, Boyd CJ. Trends in medical and nonmedical use of prescription opioids among US adolescents: 1976-2015. Pediatrics. 2017;139(4):e20162387. doi:10.1542/peds.2016-2387 28320868 PMC5369669

[3] Tomaszewski DM, Arbuckle C, Yang S, Linstead E. Trends in opioid use in pediatric patients in US emergency departments from 2006 to 2015. JAMA Netw Open. 2018;1(8):e186161. doi:10.1001/jamanetworkopen.2018.6161 30646317 PMC6324333

[4] Hudgins JD, Porter JJ, Monuteaux MC, Bourgeois FT. Trends in opioid prescribing for adolescents and young adults in ambulatory care settings. Pediatrics. 2019;143(6):e20181578. doi:10.1542/peds.2018-1578 31138669

[5] Winters KC, Arria A. Adolescent brain development and drugs. Prev Res. 2011;18(2):21-24. 22822298 PMC3399589

[6] Squeglia LM, Jacobus J, Tapert SF. The influence of substance use on adolescent brain development. Clin EEG Neurosci. 2009;40(1):31-38. doi:10.1177/155005940904000110 19278130 PMC2827693

[7] McCabe SE, West BT, Morales M, Cranford JA, Boyd CJ. Does early onset of non-medical use of prescription drugs predict subsequent prescription drug abuse and dependence? Results from a national study. Addiction. 2007;102(12):1920-1930. doi:10.1111/j.1360-0443.2007.02015.x 17916222 PMC2377405

[8] Dowell D, Haegerich TM, Chou R. CDC guideline for prescribing opioids for chronic pain—United States, 2016. MMWR Recomm Rep. 2016;65(1):1-49. doi:10.15585/mmwr.rr6501e1 26987082

[9] Matson KL, Johnson PN, Tran V, Horton ER, Sterner-Allison J; Advocacy Committee on behalf of Pediatric Pharmacy Advocacy Group. Opioid use in children. J Pediatr Pharmacol Ther. 2019;24(1):72-75. doi:10.5863/1551-6776-24.1.72 30837819 PMC6397009

[10] Phillips JK, Ford MA, Bonnie RJ, eds. Committee on Pain Management and Regulatory Strategies to Address Prescription Opioid Abuse. National Academies Press (US); 2017.

[11] Volkow ND, McLellan AT. Opioid abuse in chronic pain—misconceptions and mitigation strategies. N Engl J Med. 2016;374(13):1253-1263. doi:10.1056/NEJMra1507771 27028915

[12] Compton WM, Thomas YF, Stinson FS, Grant BF. Prevalence, correlates, disability, and comorbidity of DSM-IV drug abuse and dependence in the United States: results from the national epidemiologic survey on alcohol and related conditions. Arch Gen Psychiatry. 2007;64(5):566-576. doi:10.1001/archpsyc.64.5.566 17485608

[13] McCabe SE, Veliz P, Schulenberg JE. Adolescent context of exposure to prescription opioids and substance use disorder symptoms at age 35: a national longitudinal study. Pain. 2016;157(10):2173-2178. doi:10.1097/j.pain.0000000000000624 27227693 PMC5028228

[14] McCabe SE, West BT, Teter CJ, Boyd CJ. Medical and nonmedical use of prescription opioids among high school seniors in the United States. Arch Pediatr Adolesc Med. 2012;166(9):797-802. doi:10.1001/archpediatrics.2012.85 22566521 PMC3416923

[15] Miech R, Johnston L, O’Malley PM, Keyes KM, Heard K. Prescription opioids in adolescence and future opioid misuse. Pediatrics. 2015;136(5):e1169-e1177. doi:10.1542/peds.2015-1364 26504126 PMC4834210

[16] Boyd CJ, McCabe SE, Cranford JA, Young A. Adolescents’ motivations to abuse prescription medications. Pediatrics. 2006;118(6):2472-2480. doi:10.1542/peds.2006-1644 17142533 PMC1785364

[17] Harbaugh CM, Lee JS, Hu HM, . Persistent opioid use among pediatric patients after surgery. Pediatrics. 2018;141(1):e20172439. doi:10.1542/peds.2017-2439 29203521 PMC7053700

[18] Kelley-Quon LI, Cho J, Strong DR, . Association of nonmedical prescription opioid use with subsequent heroin use initiation in adolescents. JAMA Pediatr. 2019;173(9):e191750. doi:10.1001/jamapediatrics.2019.1750 31282942 PMC6618794

[19] Bohnert AS, Valenstein M, Bair MJ, . Association between opioid prescribing patterns and opioid overdose-related deaths. JAMA. 2011;305(13):1315-1321. doi:10.1001/jama.2011.370 21467284

[20] Han B, Compton WM, Jones CM, Cai R. Nonmedical prescription opioid use and use disorders among adults aged 18 through 64 years in the United States, 2003-2013. JAMA. 2015;314(14):1468-1478. doi:10.1001/jama.2015.11859 26461997

[21] Miller M, Barber CW, Leatherman S, . Prescription opioid duration of action and the risk of unintentional overdose among patients receiving opioid therapy. JAMA Intern Med. 2015;175(4):608-615. doi:10.1001/jamainternmed.2014.8071 25686208

[22] Kirson NY, Shei A, Rice JB, . The burden of undiagnosed opioid abuse among commercially insured individuals. Pain Med. 2015;16(7):1325-1332. doi:10.1111/pme.12768 25929289

[23] Hadland SE, Wharam JF, Schuster MA, Zhang F, Samet JH, Larochelle MR. Trends in receipt of buprenorphine and naltrexone for opioid use disorder among adolescent and young adults, 2001–2014. JAMA Pediatr. 2017;171(8):747-755. doi:10.1001/jamapediatrics.2017.0745 28628701 PMC5649381

[24] Edlund MJ, Forman-Hoffman VL, Winder CR, . Opioid abuse and depression in adolescents: results from the National Survey on Drug Use and Health. Drug Alcohol Depend. 2015;152:131-138. doi:10.1016/j.drugalcdep.2015.04.010 25981310

[25] Boyd CJ, Young A, McCabe SE. Psychological and drug abuse symptoms associated with nonmedical use of opioid analgesics among adolescents. Subst Abus. 2014;35(3):284-289. doi:10.1080/08897077.2014.928660 24905351 PMC4243924

[26] Hoppe JA, Kim H, Heard K. Association of emergency department opioid initiation with recurrent opioid use. Ann Emerg Med. 2015;65(5):493-499.e4. doi:10.1016/j.annemergmed.2014.11.015 25534654

[27] DePhillips M, Watts J, Lowry J, Dowd MD. Opioid prescribing practices in pediatric acute care settings. Pediatr Emerg Care. 2019;35(1):16-21. doi:10.1097/PEC.0000000000001239 28719481

[28] Henke RM, Tehrani AB, Ali MM, . Opioid prescribing to adolescents in the United States from 2005 to 2016. Psychiatr Serv. 2018;69(9):1040-1043. doi:10.1176/appi.ps.201700562 29983109

