# Supplementary material for: Trajectories of Opioid Use Following First Opioid Prescription in Opioid-Naive Youths and Young Adults
Source: JAMA Netw Open. 2021 Apr 22;4(4):e214552. doi: 10.1001/jamanetworkopen.2021.4552 (PMC8063062; doi:10.1001/jamanetworkopen.2021.4552)
Supplement: Supplement. — eFigure 1. Cohort Creation for Trajectory Models eTable. ICD 9 and 10 Codes Used to Define Patient Comorbidities eFigure 2. Four-Group Trajectory Model Showing Opioid Fills in 12 Months Following First Opioid Prescription [file jamanetwopen-e214552-s001.pdf]

# Supplemental Online Content

Wilson JD, Abebe KZ, Kraemer K, et al. Trajectories of opioid use following first opioid prescription in opioid-naive youths and young adults. *JAMA Netw Open.* 2021;4(4):e214552. doi:10.1001/jamanetworkopen.2021.4552

**eFigure 1.** Cohort Creation for Trajectory Models

**eTable.** ICD 9 and 10 Codes Used to Define Patient Comorbidities

**eFigure 2.** Four-Group Trajectory Model Showing Opioid Fills in 12 Months Following First Opioid Prescription

This supplemental material has been provided by the authors to give readers additional information about their work.

© 2021 Wilson JD et al. *JAMA Network Open.*

**eFigure 1. Cohort Creation for Trajectory Models**

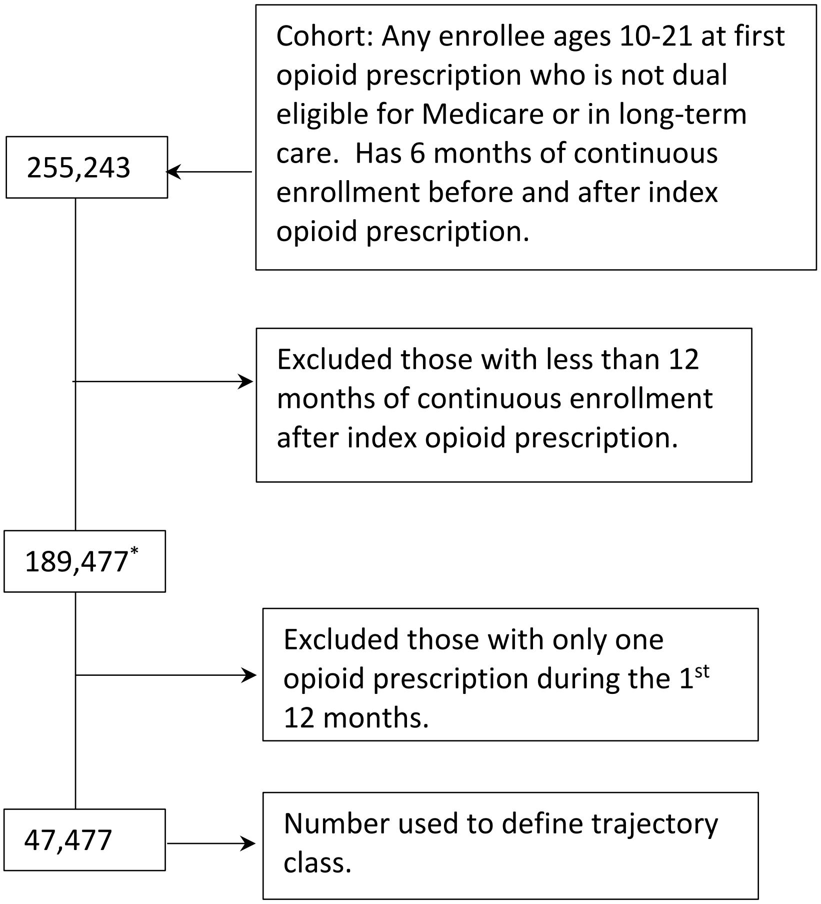


*131,234 of these enrollees included in multinomial regression to calculate odds of trajectory membership or being in single prescription group. n=54391 excluded for missing provider specialty codes; 3852 excluded for missing other variables or covariates included in the regression.

© 2021 Wilson JD et al. *JAMA Network Open.*

**eTable. ICD 9 and 10 Codes Used to Define Patient Comorbidities**

| **Variable** | Related major code groupings (included all related subcodes) |
|---|---|
| **Depression** | ICD 9: 296.20, 296.21, 296.22, 296.23, 296.24, 296.25, 296.26, 296.30, 296.31, 296.32, 296.33, 296.34, 296.35, 296.36, 300.4, 311<br>ICD 10: F32 and F33 and all subcodes |
| **Anxiety** | ICD 9: 300.02, 300.01, 300.3, 309.81, 308.3, 300.23, 300.2, 291.89, 292.89, 309.21, 300<br>ICD 10: F41, F43, F43.2, F48.8, F45, F93, F41.0, F41.1, F4.3, F41.8, F41.9 |
| **Attention Deficit Disorder** | ICD 9: 314.00, 314.01, 314.8<br>ICD 10: 90.9, 90.2, 90.8, 90.1, 41.840 |
| **Substance use disorder** | ICD9: 303, 303.9 [0-3] ,304.0[0-3], 304.1[0-3], 304.2[0-3], 304.3[0-3], 304.4[0-3], 304.5[0-3], 304.6[0-3], 304.9, 304.8, 305.0[0-3], 305.1[0-3], 305.2[0-3], 305.3[0-3], 305.4[0-3], 305.5[0-3], 305.6[0-3], 305.7[0-3], 305.8[0-3], 305.9[0-3]<br><br>ICD10: F10, F11, F12, F13, F14, F15, F16, F17, F18, F19 (and all subcodes) |

© 2021 Wilson JD et al. *JAMA Network Open.*

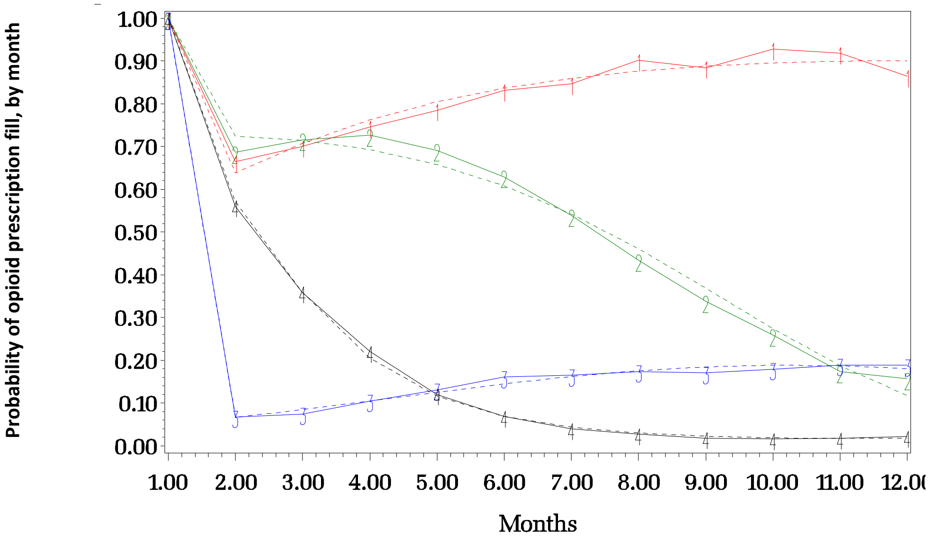


**eFigure 2. Four-Group Trajectory Model Showing Opioid Fills in 12 Months Following First Opioid Prescription.**

Y-axis represents proportion of each trajectory group filling an opioid prescription for a given month, which is listed on x-axis. Month 1 is month of first opioid prescription. Dotted line represents estimated trajectory and solid line represents observed data. Color and number reflects group membership.

**Four-group model:** Group 1 “persistently high” = red, n= 780 (1.8%); Group 2 “lagged decrease” = green, n=1124 (2.7%); Group 3 “rapid decline”, = blue, n=29,826 (63.2%); Group 4 “low” = black, n=15,744 (32.3%).

.

© 2021 Wilson JD et al. *JAMA Network Open.*
